# Potent anti-inflammatory activity of the lectin-like domain of TNF in joints

**DOI:** 10.3389/fimmu.2022.1049368

**Published:** 2022-10-31

**Authors:** Ana Carolina Matias Dinelly Pinto, Rodolfo de Melo Nunes, Igor Albuquerque Nogueira, Bernhard Fischer, Rudolf Lucas, Virgínia Claudia Carneiro Girão-Carmona, Vivian Louise Soares de Oliveira, Flavio Almeida Amaral, Georg Schett, Francisco Airton Castro Rocha

**Affiliations:** ^1^ Departamento de Medicina Interna, Faculdade de Medicina, Universidade Federal do Ceará, Fortaleza, Brazil; ^2^ Apeptico Forschung und Entwicklung, Vienna, Austria; ^3^ Vascular Biology Center, Department of Pharmacology and Toxicology, Division of Pulmonary and Critical Care Medicine, Medical College of Georgia at Augusta University, Augusta, GA, United States; ^4^ Departamento de Morfologia, Faculdade de Medicina, Universidade Federal do Ceará, Fortaleza, Brazil; ^5^ Departamento de Bioquímica e Imunologia, Universidade Federal de Minas Gerais, Belo Horizonte, Brazil; ^6^ Department of Internal Medicine 3, Rheumatology and Immunolgy, Friedrich-Alexander-University Erlangen-Nürnberg, Erlangen, Germany

**Keywords:** arthritis, neutrophils, cytokines, inflammation, tumor necrosis factor

## Abstract

In view of the crucial role of tumor necrosis factor (TNF) in joint destruction, TNF inhibitors, including neutralizing anti-TNF antibodies and soluble TNF receptor constructs, are commonly used therapeutics for the treatment of arthropathies like rheumatoid arthritis (RA). However, not all patients achieve remission; moreover, there is a risk of increased susceptibility to infection with these agents. Spatially distinct from its receptor binding sites, TNF harbors a lectin-like domain, which exerts unique functions that can be mimicked by the 17 residue solnatide peptide. This domain binds to specific oligosaccharides such as *N*′*N*′-diacetylchitobiose and directly target the α subunit of the epithelial sodium channel. Solnatide was shown to have anti-inflammatory actions in acute lung injury and glomerulonephritis models. In this study, we evaluated whether the lectin-like domain of TNF can mitigate the development of immune-mediated arthritis in mice. In an antigen-induced arthritis model, solnatide reduced cell influx and release of pro-inflammatory mediators into the joints, associated with reduction in edema and tissue damage, as compared to controls indicating that TNF has anti-inflammatory effects in an acute model of joint inflammation *via* its lectin-like domain.

## Introduction

Tumor necrosis factor (TNF) is a major cytokine, existing as a transmembrane and soluble ligand, which is involved in various acute and chronic inflammatory diseases. Robust evidence has linked overexpression of TNF to arthritis severity in animal models. TNF inhibitors (TNFi), including neutralizing monoclonal anti-TNF antibodies and soluble TNF receptor constructs, have revolutionized the treatment of rheumatoid arthritis (RA) and spondyloarthritis (SpA) in the clinical setting. TNF is not only a main mediator of pro-inflammatory mediator release, cell migration, and pain development, but the cytokine also activates enzymes that are involved in joint destruction, by eroding cartilage while inducing inflammatory bone resorption (1). Paradoxically, TNF was demonstrated to provide benefit in SpA patients where bone formation rather than erosion ensues, prompting syndesmophyte formation and joint fusion secondary to inappropriate bone formation (2). After cleavage by ADAM17 [A disintegrin and metalloproteinase 17, a.k.a. TNF-alpha converting enzyme (TACE)], the transmembrane form of TNF (tmTNF) is released, generating the soluble TNF (sTNF) ligand. Both TNF forms can bind to both TNF receptors I (RI, 55 kDa) and II (RII, 75 kDa)—with tmTNF having higher affinity for TNF-RII than sTNF—thereby leading to cell death or inflammation (3–6). A marked increase in tmTNF was detected in models of SpA, as compared to RA, correlating with a decrease in the enzymatic activity of ADAM17 (7). These findings suggest that the two TNF forms can induce different activities, despite sharing the same molecular structure and a comparable avidity for TNF RI (7).

In sharp contrast to its deleterious role in numerous pathophysiological phenomena, including the above-described arthropathies, TNF also represents a major component of the first-line defense against invasion by intracellular microorganisms, in addition to its beneficial function in tumor surveillance (8).

The lectin-like domain of TNF, which recognizes specific sugar ligands, such as *N,N*′-diacetylchitobiose (NAc) and branched trimannoses, is spatially distinct from its receptor binding sites (9, 10). Mapping of the lectin-like domain of TNF was achieved by means of studying the lytic effect of sTNF against bloodstream forms of African trypanosomes (10–12), resulting in the generation of the TNF-derived peptide solnatide (a.k.a. TIP peptide, AP301) with sequence CGQRETPEGAEAKPWYC. Although these protozoan parasites lack TNF receptors, TNF was able to kill the parasites *via* a mechanism involving its lectin-like domain, which can be specifically blocked by the oligosaccharide NAc (10). We have also shown that TNF prevents the formation of *Candida albicans* biofilms, an effect that could be blocked by adding a TNF monoclonal antibody but not the soluble TNF receptor construct etanercept, the latter of which was shown not to interfere with the activity of the lectin-like domain (13). Interestingly, the isolated addition of NAc specifically blocked the TNF effect on *Candida biofilm* formation, strongly suggesting that TNF was operating *via* its lectin-like domain (14).

More recently, it was demonstrated that solnatide improves lung function in acutely inflamed lungs in both animal models of acute respiratory distress syndrome (ARDS) and in ARDS patients (15–18) upon binding to the α subunit of the epithelial sodium channel (ENaC), which can be expressed in both epithelial and endothelial cells (16, 19–22). Solnatide (a.k.a. TIP peptide, AP301) has also been shown to protect kidneys and to exert potent anti-inflammatory activity in an experimental glomerulonephritis model (19). Thus, in addition to different effects of the cytokine dependent on whether tmTNF or sTNF is predominant, TNF might have other activities that are independent of binding to its specific TNF receptors.

Chondrocytes, osteoblasts, and mesenchymal stem cells have been shown to express ENaC-a, to which solnatide binds (22–25). Although the benefit of TNF inhibitors—which block the binding of TNF ligands to their receptors—in treating arthropathies is undisputable, not all patients achieve remission, and there is a risk of increased susceptibility to infection (26), in view of the important role of TNF receptors in host immune defense to invading pathogens. Moreover, some SpA patients hardly display clinically relevant benefit from TNFi administration (27). Hence, in this study, we investigated whether TNF-receptor-independent activities of TNF, such as those mediated by the TNF-derived solnatide peptide, which mimics the lectin-like domain, are protective in an immune-mediated arthritis [methylated bovine serum albumin (mBSA)] mouse model.

## Methods

### Reagents

All reagents were purchased from Sigma Chem Co., São Paulo, Brazil, unless stated otherwise. Solnatide was kindly donated by Apeptico Forschung und Entwicklung, Vienna, Austria, and was dissolved at the time of use using Roswell Park Memorial Institute (RPMI) 1640 supplemented with L-glutamine.

### Animals

A total of 42 Balb-C mice of either sex (25–30 g) were provided by the central animal facilities of our institution. Animals were housed in cages (six per cage) in temperature-controlled rooms with a 12-h light/dark cycle with free access to water and food. At the start of any experiments, mice were 2.5 months of age. All animal procedures and experimental protocols were approved by our local ethics committee on animal experimentation, which follows the recommendations of the Brazilian Council on Animal Experimentation (CONCEA) (protocol number 113/07). All efforts were made to minimize animal suffering and the number of animals used.

### mBSA arthritis

Groups of mice received either 500 μg subcutaneous (s.c.) methylated bovine serum albumin (mBSA) s.c. mixed with Freund’s complete adjuvant (immunized) or incomplete Freund’s adjuvant [false-immunized (FI)], followed by a booster injection 7 days later. Twenty-one days following this immunization process, mice received intra-articular (i.a.) 90 μg mBSA. Groups were then sacrificed 7 h (acute phase) or 3 days (chronic phase) following the i.a. mBSA challenge. All injections were done under xylazine (10 mg/kg)/ketamine (80 mg/kg) intra-peritoneal (i.p.) anesthesia, and sacrifice was done under terminal anesthesia.

### Treatments

In order to investigate the effect of blocking the endogenous lectin-like domain of TNF, groups of mice received 100 μg of NAc in 10 μl saline i.a. or 10 μl saline, 30 min prior to mBSA i.a. In a strategy to evaluate a possible preventive local anti-inflammatory activity of solnatide, groups of naive mice subjected to mBSA arthritis received 10–20 μg of 10 μl i.a. solnatide solutions. As a local therapeutic strategy, another group of mice received 10 μg/10 μl solnatide or 10 μl saline given i.a. 2 days after i.a. challenge with mBSA followed by sacrifice, under terminal anesthesia, after 24 h. In a last set of experiments, as a systemic therapeutic strategy, another group of mice subjected to mBSA arthritis received intravenous (i.v.) solnatide (1–10 μg) immediately prior to i.a. challenge with mBSA.

### Assessment of pain behavior and knee joint swelling

Nociceptive behavior was assessed using the electronic pressure meter nociception paw test by an observer blinded to group allocation (28). Animals were placed in acrylic cages (12 × 10 × 17 cm high) with a wire grid floor, 15 min before the beginning of the tests, in a quiet room. Stimulations were performed only when animals were quiet, without exploratory, urination, or defecation movements and not resting on their paws. The electronic pressure meter consists of a hand-held force transducer fitted with a polypropylene tip (Electronic von Frey aesthesiometer, Insight Equipamentos Científicos Ltda., Brasil). The polypropylene tip was applied perpendicularly to one of the five distal footpads of the right hind paw. The intensity of the stimulus was automatically recorded when the paw was withdrawn. The test was repeated three times, until less than 1 g difference between measurements was obtained. Results were expressed as the mean value of three withdrawal threshold measurements (g). The diameter of the joints was measured with a caliper (mm), with the whole knee joint swelling determined as the increase in joint diameter. Results were expressed as the Δ (mean variation of three measurements for each joint) relative to baseline.

### Assessment of cell influx and inflammatory mediators in joint exudates

Animals were sacrificed under anesthesia, and the synovial cavity of the knee joints was washed with 0.05 ml saline containing 10 mmol/L EDTA. Joint washes were collected by aspiration, and total cell counts were performed using a Neubauer chamber. Differential cell counts were performed using the panoptic Instant Prov™ staining kit (New ProvBrasil™). After centrifuging (500 g/10 min), the supernatants were stored at −80°C until used for measuring the concentrations of interleukin (IL)-1β, IL-6, CCL-2, and CXCL1 using commercially available kits (R & D Systems, São Paulo, Brazil).

### Histopathology

Knee joint tissues were excised for the histological study. After fixation in 10% v/v formaldehyde solution and decalcification (5% v/v formic acid in 10% v/v formaldehyde solution), the whole joint, comprising the distal femoral and proximal tibial extremities, was processed for paraffin-embedding and staining with hematoxylin-eosin (HE) and safranin-O. Analysis was expressed as one result/sample. Semi-quantitative histopathological evaluations were performed by an independent observer (VCCG) blinded to group allocation considering synovial proliferation and cell infiltration and glycosaminglycan content (safranin staining intensity), ranging from 0 to 3 (0, absent; 1, mild; 2, moderate; 3, severe). Results were expressed as the median value for each group of four animals.

### Statistical analysis

Results were presented as means ± SD for pain behavior and cell counts in joint washings or medians for histology of measurements made on at least three animals in each group. Differences between means and medians were compared using Student’s “t” or Mann–Whitney tests, respectively; *p* < 0.05 was considered as significant.

## Results

### Effect of the lectin-like domain inhibitor *N,N*′-diacetylchitobiose in immune-mediated arthritis

**Figure 1:**
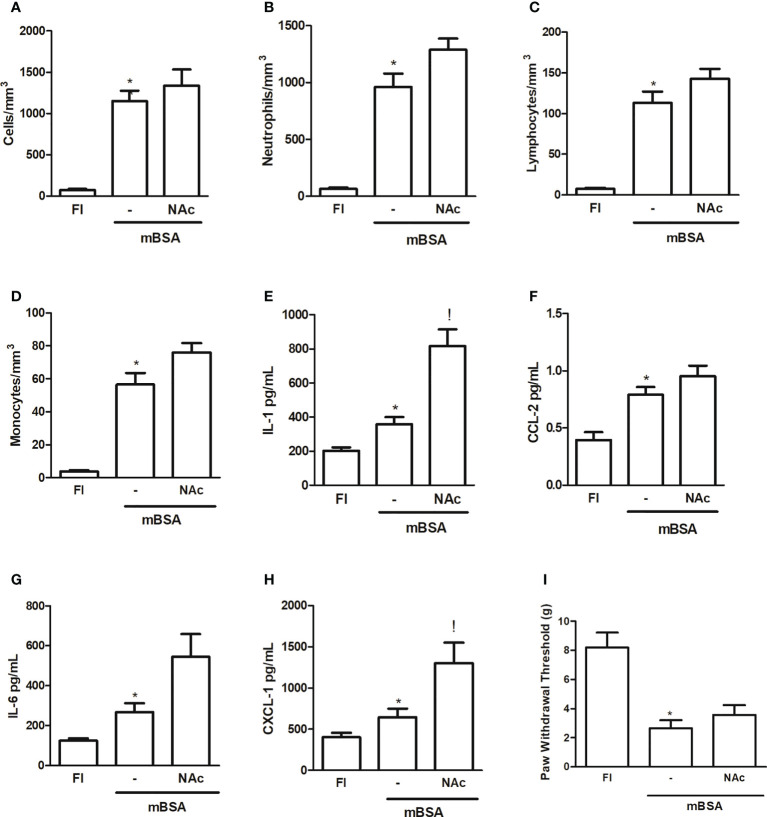
Mice subjected to mBSA arthritis received i.art. *N*-*N*′-diacethylchitobiose (NAc; 100 µg/10 µl) or saline (−) 30 min prior to challenge with mBSA. Cell counts and cytokine and chemokine levels were assayed in joint washes collected after 7 h; FI stands for control, false-immunized animals; *p<0.05 compared to FI;!p<0.05 compared to mBSA, using Student’s “t” test (n≥3 animals/group). **(A–D)** Total and differential cell counts; **(E–G)** interleukin (IL-1), IL-6, CXCL-1, and CCL-2 levels; **(I)** hypernociception measured using von Frey’s test.

**Figure 1** shows that injection of NAc, which binds to the lectin-like domain of TNF (9, 10) into the joints of mice subjected to mBSA arthritis, caused a trend to increased cell counts—albeit not statistically significant—measured after 7 h in joint exudates, with a predominance (>85%) of polymorphonuclear cells (**Figures 1A–D**). This strategy aimed to determine the effect of specifically blocking the lectin-like domain of TNF released following development of mBSA arthritis. The injection of NAc was also associated with an increase in the levels of pro-inflammatory cytokines and chemokines assayed, although only the increase in IL-1 and CXCL1 reached statistical significance, as compared to levels in animals subjected to mBSA arthritis that received saline (**Figures 1E–G**). Joint hyper-nociception was not altered in mice subjected to mBSA arthritis that received NAc (**Figure 1I**).

### Effect of a prophylactic intra-articular administration of solnatide in immune-mediated arthritis

**Figure 2:**
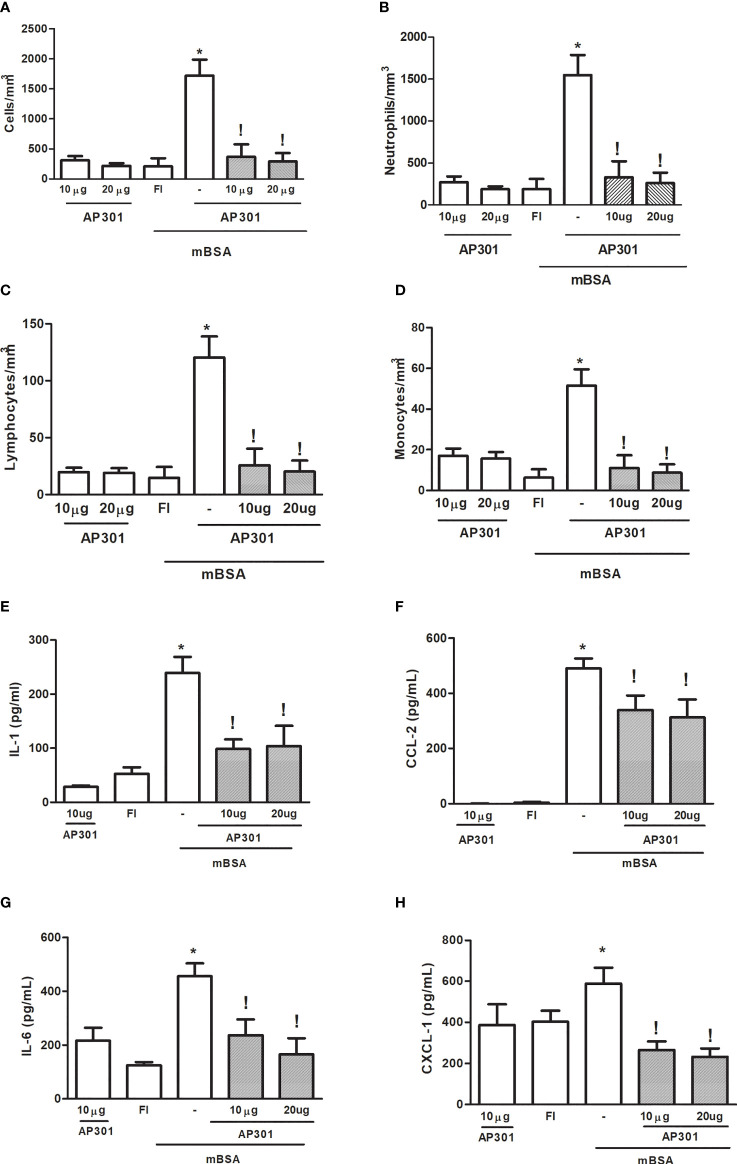
Mice subjected to mBSA arthritis received. i.art. solnatide (AP 301) or saline (−) 30 min prior to challenge with mBSA. Cell counts and cytokine and chemokine levels were assayed in joint washes collected after 7 h. FI stands for control, false-immunized animals; *p<0.05 compared to FI;!p<0.05 compared to mBSA, using Student’s “t” test (n≥3 animals/group). **(A–D)** Total and differential cell counts; **(E–H)** interleukin (IL-1), IL-6, CXCL-1, and CCL-2 levels.

Administration of solnatide (10 or 20µg) into the joints of naive mice promoted a mild, acute cell influx, with predominance (>85%) of polymorphonuclear cells, which was similar to that observed in FI animals (**Figures 2A–D**). Similarly, cytokine and chemokine levels in joint exudates of naive mice that received solnatide did not significantly differ from those observed in FI animals, used as controls (**Figures 2E–H**). By contrast, the treatment of mice subjected to mBSA arthritis with solnatide significantly reduced cell counts in joint exudates, measured at 7 h after intra-articular challenge (**Figures 1A–D**). Additionally, levels of pro-inflammatory cytokines and chemokines were also significantly reduced in mice subjected to mBSA arthritis that were treated with solnatide (**Figures 2E–H**).

### Effect of a systemic administration of solnatide in immune-mediated arthritis

**Figure 3:**
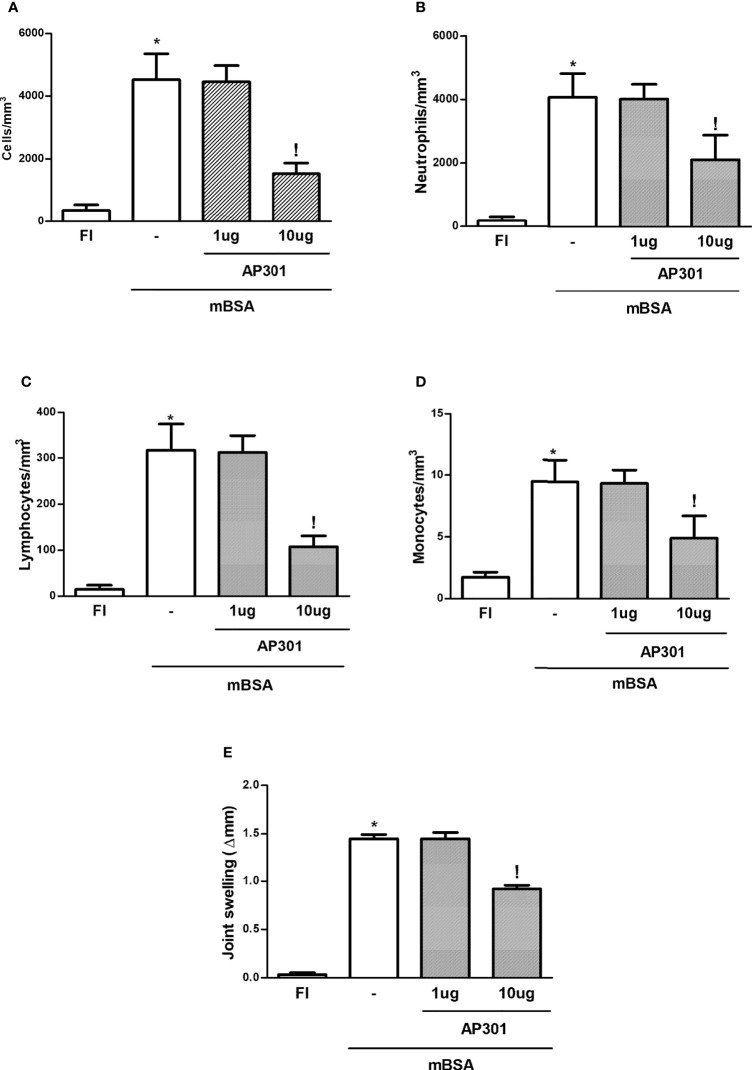
Mice subjected to mBSA arthritis received i.v. solnatide (AP 301) or saline (−) just prior to challenge with mBSA. Joint volume and cell counts were assessed with calipers or in joint washes, respectively. FI stands for control, false-immunized animals; *p<0.05 compared to FI;!p<0.05 compared to mBSA, using Student’s “t” test (n≥3 animals/group). **(A–D)** Total and differential cell counts; **(E)** Δ paw volume relative to baseline.

Similar to what was seen with the local prophylactic strategy mentioned above, systemic (i.v.) administration of AP301 led to a significant decrease in joint edema and of the acute cell influx into joint exudates, with a decrease in polymorphonuclear cell, lymphocyte, and mononuclear cell counts (**Figures 3A–E**).

### Effect of a therapeutic intra-articular administration of solnatide in immune-mediated arthritis

**Figure 4:**
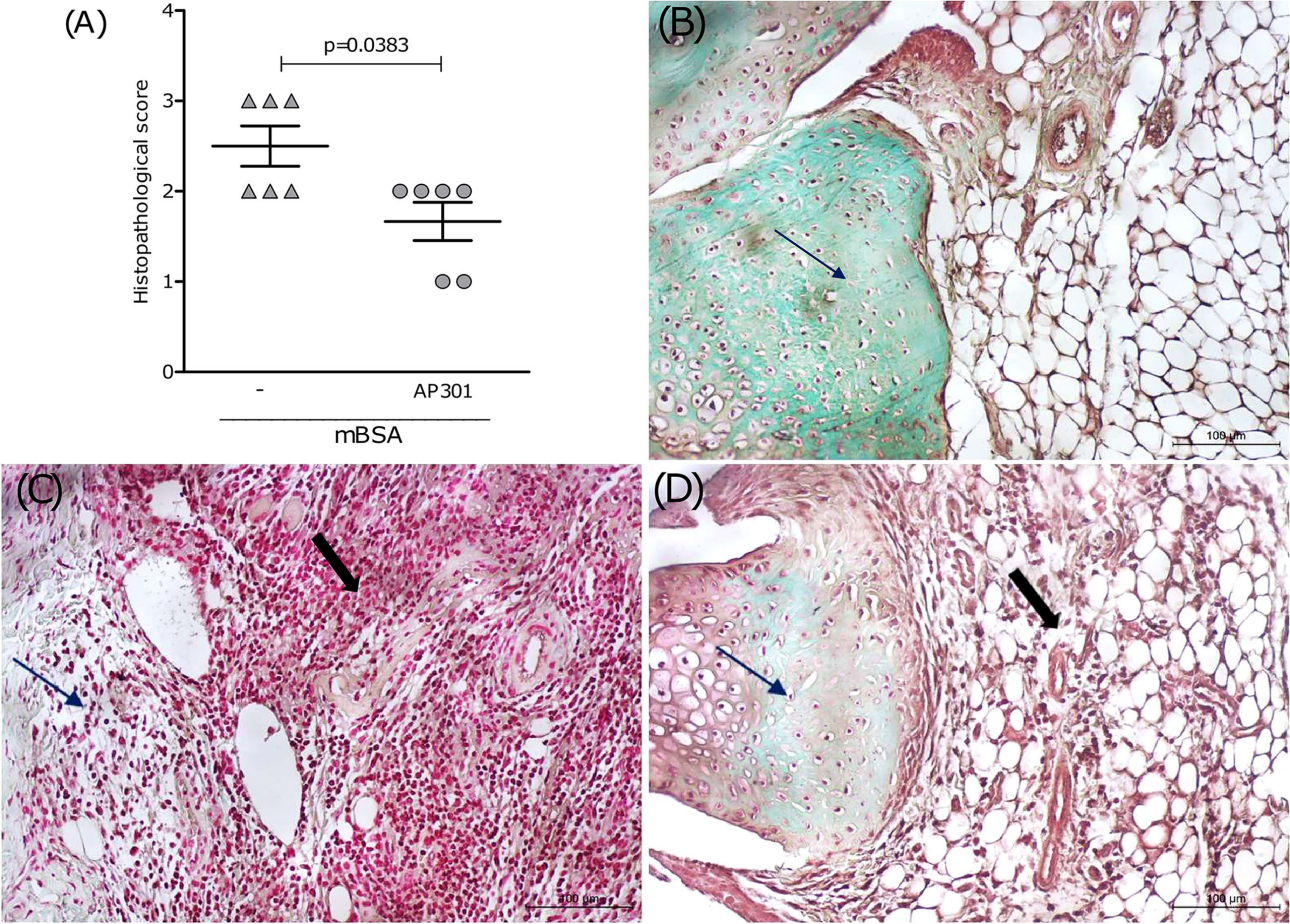
Mice subjected to mBSA arthritis received i.art. solnatide (AP 301) or saline (−) 2 days after challenge with mBSA being sacrificed after 24 h; **(A)** score (medians) of HE-stained whole joints compared using Mann–Whitney. Representative illustrations of synovium and cartilage (arrows) stained with safranin O and synovial cell influx (solid arrows); **(B)** naive joint; **(C)** mBSA (−); **(D)** mBSA treated with AP301; original 200×.

In order to evaluate the therapeutic potential of the lectin-like domain of TNF, a group of mice subjected to mBSA arthritis received solnatide (10 or 20 µg i.a.) 2 days after intra-articular challenge with mBSA. These mice were sacrificed after 24 h, and the joints were excised for histopathological examination. Results in **Figure 4A** show a significant reduction in histological scores, followed by representative illustrations of histological sections. Arrows show that staining with safranin O, indicative of glycosaminoglycan content in the cartilage, is reduced in mice subjected to mBSA arthritis treated with saline (**Figure 4C**), as compared to the joint of a naive animal (**Figure 4B**). **Figure 4D** illustrates a partial restoration of staining of the glycosaminoglycans of the joint cartilage in a sample from the group that received solnatide, which means reduced cartilage damage. An extensive cell infiltration and synovial proliferation can also be seen in the sample from animals subjected to mBSA arthritis treated with saline (**Figure 4C**), which is significantly reduced in the samples from mice treated with solnatide (**Figure 4D** and **Table 1**).

**Table 1:** Histopathological analysis of the effect of solnatide in mBSA arthritis.

	Cell influx	Synovial proliferation	Cartilage damage	Total Score
Naive	0 (0 -0)	0 (0-1)	0 (0-0)	0 (0-0)
NT	2.5 (2-3)	1 (1-2)	2 (2-3)	2.5 (2-3)
Solnatide	1 (1-2)	1 (0-1)	1 (1-2)	2.5 (1-2)
P value*	0.0086	0.0256	0.0208	0.0383

Mice subjected to mBSA arthritis received i.art. solnatide or saline (NT) 2 days after challenge with mBSA being sacrificed after 24h; a) Score (medians) of HE and Safranin-O stained whole joints compared using Mann-Whitney; n=6 animals/group; NT, non-treated; p value comparing NT vs Solnatide.

## Discussion

The development of a synthetic peptide, solnatide (a.k.a. TIP peptide, AP301), which encompasses the amino acid sequence of the lectin-like domain of TNF, is a valuable tool to dissect TNF receptor-dependent from TNF receptor-independent effects. Similar to what happens in the above-described arthropathies, activation of TNF RI has been shown to be crucial to TNF effects in immune glomerulonephritis (29), where it mediates the recruitment of monocytes and activation of mesangial and endothelial cells and podocytes, at least partially *via* the p38 MAP kinase pathway (19, 30–32). Administration of solnatide (i.p.) significantly reduced inflammation in an experimental glomerulonephritis model, induced by nephrotoxic serum, as it was associated with the reduction in the release of IL-1β and IL-6 and of the chemokines macrophage chemotactic protein (MCP)-1 and keratinocyte-derived chemokine (KC) (19). Similarly, a previous study showed that intratracheal administration of solnatide to rats subjected to acute hypobaric hypoxia followed by exercise reduced pulmonary edema, decreasing alveolar hemorrhage and lung damage. This protective effect was associated with the decreased release of IL-1, IL-6, TNF, and IL-8 into the bronchoalveolar lavage fluid (33).

Our present data for the first time demonstrate that solnatide significantly reduces inflammatory cell infiltration and joint damage in an immune-mediated (mBSA) experimental arthritis model in mice. The effect of solnatide was observed using both prophylactic and therapeutic strategies and following local (intra-articular) and systemic (intravenous) administration. In keeping with data from other studies, solnatide actions were also associated with a reduction in pro-inflammatory cytokine (IL-1β and IL-6) and chemokine (CCL2 and CXCL-1) levels in joint exudates.

The mechanism of action of solnatide involves binding specifically to the C-terminal domain of the α-subunit of the epithelial sodium channel (ENaC) (15, 21, 22). ENaC expression has been demonstrated in a range of epithelial and endothelial cells (15, 16). In joints, ENaC is expressed in chondrocytes and osteoblasts (24, 25). Our observation that solnatide was effective not only when injected locally but also following a systemic prophylactic administration led us to speculate that it operates *via* resident cells inside the joint. In addition to a rich blood supply, with fenestrated capillaries, the synovial tissue harbors type I, fibroblast-like, and type II, macrophage-like synoviocytes (34). Although chondrocytes and osteoblasts were shown to express ENaC receptors, it is likely that endothelial cells and type II synoviocytes, which were also shown to express ENaC (19, 25), are the primary targets of this peptide following intra-articular injection rather than infiltrating cells, which gain access to the joint approximately 4 h following joint challenge. Additionally, it has been shown that infiltrating leukocytes do not express ENaC (35, 36). Coupling of solnatide to ENaC channels in endothelial cells could reduce inflammation, thereby reducing local edema, as observed in our study. In accordance with our observations in other models of inflammation (19), it is likely that binding of solnatide to ENaC expressed in type II (macrophage-like) synoviocytes would downregulate the release of pro-inflammatory chemokines and cytokines, thereby reducing cell recruitment and the subsequent release of additional inflammatory mediators. As a result, administration of solnatide would lead to an earlier resolution of inflammation and joint destruction in our immune-mediated arthritis model, as indicated by our present data. A previous study showing that solnatide activates ENaC in mice lacking both TNF receptors (37) and the recent demonstration that depletion of the α subunit of the ENaC blunted the protective effect of solnatide in a TNF-induced inflammation in glomerular endothelial cells (19) led us to propose that there is a high likelihood that solnatide exerts its anti-inflammatory effects in mBSA arthritis *via* a mechanism independent of the classical TNF receptors but dependent upon the interaction between the lectin-like domain of the cytokine mimicked by solnatide and ENaC. This is substantiated by our observation that treatment with NAc, an oligosaccharide blocking the lectin-like activity of TNF, aggravates pathology.

In summary, these data show that solnatide, a synthetic peptide mimicking the lectin-binding domain of TNF, reduces inflammation in an immune-mediated arthritis model. The anti-inflammatory activity of solnatide is associated with the reduction in joint damage. Since local therapeutic applications of solnatide have already been carried out in humans and have shown to dampen lung inflammation, there is a strong rationale to use solnatide as local treatment in inflammatory arthritis to achieve resolution of arthritis.

## Data availability statement

The original contributions presented in the study are included in the article/supplementary material. Further inquiries can be directed to the corresponding author.

## Ethics statement

The animal study was reviewed and approved by Brazilian Council on Animal Experimentation (CONCEA).

## Author contributions

FR, FA and GS contributed to the conception of the protocol. FR, RL, AP, IN, VG-C and VO performed animal studies, including histology reading (VG-C). FR, FA and VO performed cytokine measurements. FR, FA, RL, BF and GS wrote the manuscript. All authors contributed to the article and approved the submitted version.

## Funding

RL received funding from NIH/NHLBI grant R01 HL138410A; FAA received funding from Fundação de Amparo à Pesquisa de Minas Gerais (FAPEMIG, #APQ-01608-17).

## Acknowledgments

We thank Conselho Nacional de Desenvolvimento Científico e Tecnológico (CNPQ)—Brazil for providing partial support for Prof. Rocha. We think Apeptico™ for kindly providing solnatide. We acknowledge partial support of this work from CNPq (CNPQ grants 313860/2021-1 and 403767/2021-0).

## Conflict of interest

We received the peptide solnatide from Apeptico as a kind donation but all experiments were run in Brazil Fortaleza and Belo Horizonte and Germany Erlangen. Author BF is an employee of APEPTICO Forschung und Entwicklung. Author RL is an inventor of patents with solnatide.

The remaining authors declare that the research was conducted in the absence of any commercial or financial relationships that could be construed as a potential conflict of interest.

## Publisher’s note

All claims expressed in this article are solely those of the authors and do not necessarily represent those of their affiliated organizations, or those of the publisher, the editors and the reviewers. Any product that may be evaluated in this article, or claim that may be made by its manufacturer, is not guaranteed or endorsed by the publisher.
